# Food Insecurity, Health, and Socioeconomic Status: Results from the University of the Basque Country, Spain

**DOI:** 10.3390/nu17081314

**Published:** 2025-04-10

**Authors:** Laura García-Iruretagoyena, Naiara Martinez-Perez, Liesbeth Colen, Miriam Baeta, Iñigo Olalde, Liv Elin Torheim, Marta Arroyo-Izaga

**Affiliations:** 1 Department of Pharmacy and Food Sciences, University of the Basque Country UPV/EHU, 01006 Vitoria-Gasteiz, Spain; 2 Department of Nursing I, University of the Basque Country UPV/EHU, 48940 Leioa, Spain; 3 BIOMICs Research Group, Microfluidics & BIOMICs Cluster, Lascaray Research Centre, University of the Basque Country UPV/EHU, 01006 Vitoria-Gasteiz, Spain; 4 Department of Agricultural Economics and Rural Development, Faculty of Agricultural Sciences, University of Göttingen, 37073 Göttingen, Germany; 5 Department of Zoology and Animal Cellular Biology, University of the Basque Country UPV/EHU, 01006 Vitoria-Gasteiz, Spain; 6 Bioaraba, BA04.03, 01006 Vitoria-Gasteiz, Spain; 7 Ikerbasque-Basque Foundation of Science, 48009 Bilbao, Spain; 8 Department of Nursing and Health Promotion, Faculty of Health Sciences, Oslo Metropolitan University (OsloMet), 0130 Oslo, Norway; livtor@oslomet.no

**Keywords:** food insecurity, higher education, health, socioeconomic status, mediation

## Abstract

**Background/Objectives:** Food insecurity (FI) is the ‘limited or uncertain availability of nutritionally adequate and safe foods’. Although the literature suggests a strong association between FI, socioeconomic status (SES), and health, the nature of their relationship is not well specified in vulnerable population groups such as university students. To address this gap, this study aimed to assess the prevalence of FI among university students, examine its association with various health outcomes, and explore the potential mediating effect of SES. **Methods:** This cross-sectional survey included a convenience sample of 394 participants from the University of the Basque Country UPV/EHU (Spain). Data on SES, demographic and lifestyle factors, and health outcomes were collected between December 2021 and January 2022, using a questionnaire developed by the Food Insecurity among European University Students during the COVID-19 Pandemic (FINESCOP) consortium. Internal consistency of the questionnaire was assessed at the UPV/EHU using Cronbach’s α. FI was measured using the Food Insecurity Experience Scale (FIES) from the FAO, which was validated through testing of Rasch model assumptions. **Results:** Overall, 19% of university students were food insecure, with 2.5% experiencing moderate and 0.8% experiencing severe FI. Adjusted linear regression models showed that FI was associated with a higher body mass index (BMI), poorer self-rated health, and worsening health during the pandemic. SES mediated the relationship between FI and health outcomes, with the strongest mediation observed for BMI (indirect association: B = 0.25, 95% CI = −0.17–0.75; total association: B = 1.85, 95% CI = 0.14–3.56; 15.3% of mediation). **Conclusions:** FI is prevalent among UPV/EHU students, and it is associated with multiple negative health outcomes, partly explained by SES. To effectively address FI, higher education institutions should consider implementing comprehensive strategies. For future research, longitudinal studies would be recommended to systematically monitor FI and examine causal relationships.

## 1. Introduction

Food insecurity (FI) is defined as “the lack of regular access to enough safe and nutritious food for normal growth and development and an active and healthy life” [1]. In 2015, the United Nations set the elimination of all forms of hunger and malnutrition worldwide by 2030 as one of its Sustainable Development Goals. However, FI remains a global issue contributing to socioeconomic health inequalities [2]. Additionally, the COVID-19 (SARS-CoV-2) pandemic further exacerbated FI in households worldwide, aggravating preexisting health disparities related to food security status [3]. In Spain, for instance, the prevalence of severe FI has doubled since the onset of the COVID-19 pandemic [4] and has worsened as a result of the armed conflict in Ukraine and climate change (causing an increase in the cost of the Basic Food Basket). This has led to the publication of a manifesto by the General Council of Official Colleges of Dietitians and Nutritionists and the Spanish Academy of Nutrition and Dietetics as a call for awareness and action to improve the detection and treatment of FI in households [5]. Despite the alarming trends, scientific research on FI within Europe remains limited [6].

In countries where food availability is not an issue, FI is often associated with numerous adverse health outcomes, including obesity and various mental health disorders [7,8,9,10]. These associations may stem from shifts toward food choices that lead to energy-dense but nutrient-poor diets, which in turn contribute to both FI and poor health outcomes such as obesity [11]. Furthermore, FI is considered a stark indicator of inequality, reflecting an individual’s socioeconomic status (SES). However, while most of the existing research on FI has primarily explored the nutritional implications, less attention has been given to the socioeconomic pathways through which FI influences health [12]. Understanding these socioeconomic pathways is crucial for public health, especially in contexts where food access and eating practices can be an indicator of socioeconomic position and/or social inclusion [13].

The conceptual explanation of how FI would have a causal mediation path via SES on health outcomes is based on Campbell’s conceptual framework for FI, its risk factors, and its consequences [14]. Campbell points out that the consequences of FI must be distinguished from the consequences of its common risk factors such as poverty and low SES. Poverty and low SES have well-documented relationships to poor health status [15]. The challenge for the field of nutritional sciences is to examine how FI interacts with SES to influence health outcomes.

In recent years, FI in higher education has gained attention [16]. University students are at a higher risk of FI compared to the general population, with average rates of 42% of FI, according to the systematic review conducted by Bruening et al. in 2017 (in which most studies used the USDA’s 10-item Adult Food Security module) [17]. The fact that university students are considered a risk group for FI has also been confirmed in more recent studies [18,19,20]. However, the literature still has very scarce information regarding FI levels among university students in European countries [21,22,23], and no data were found in relation to Spanish university students, with the exception of those from the Basque Country [24]. Various factors contribute to this vulnerability to FI among university students, including high tuition fees, increasing living costs, and limited financial support [16]. Unlike other groups, university students often lack stable employment, rely on financial aid, and have limited cooking facilities, which exacerbates their risk of FI.

Given the expansion of higher education and the growing socioeconomic diversity of the university student population, it is reasonable to assume that FI among university students has significant health consequences, which vary based on SES, although, to our knowledge, there are no publications on the influence of SES on the association between FI and adverse health outcomes in university students. To address this gap in the literature, this study aimed to (i) assess the prevalence of FI among university students of a Spanish public university during the pandemic, (ii) examine the relationships between FI and various health outcomes (specifically, body mass index (BMI) and self-reported physical and psychological health), and (iii) determine whether SES mediates the relationship between FI and health outcomes mentioned in the previous objective. We hypothesized that (i) a significant proportion of university students experience FI; (ii) FI was associated with overweight/obesity (Ov/Ob), self-rated health, and health deterioration during the pandemic; and (iii) SES, specifically the educational level of parents or legal guardians (ELoP/Lg) and employment situation (ES), mediated the relationship between FI and Ov/Ob, self-rated health, and health deterioration during the pandemic. By providing an in-depth understanding of FI among university students, this research seeks to raise awareness of this critical concern and propose strategies that support those in need.

## 2. Materials and Methods

### 2.1. Study Sample

This study is part of the Food Insecurity among European University Students during the COVID-19 Pandemic (FINESCOP) project, an observational cross-sectional investigation of students enrolled in European universities. This manuscript presents part of the results registered at a Spanish public university, the University of the Basque Country UPV/EHU. To be eligible to participate in the study, respondents had to be 18 years or older, enrolled as graduate or postgraduate students (Master’s level or higher), and have Internet access, since the survey was administered online. No student was excluded, following the example of previous studies [16], in order to compare results. Details on sample size estimation and survey administration have already been described [24]. The sample size was estimated using the Epidat 3.0 program [25], based on a total number of students enrolled during the 2020/21 academic year and the prevalence of FI reported in a similar previous study [16]. A precision level of 5%, a confidence interval (CI) of 95%, and a *p*-value of 0.05 were established. The estimated average sample size was 342 students.

The survey was conducted between December 2021 and January 2022, using the Qualtrics software (Provo, UT, USA, 2021. https://www.qualtrics.com), configured to avoid duplicate submissions. Recruitment was conducted through the university’s notice board (EHUTaula) and the UPV/EHU Sustainability Directorate’s website. Participation was anonymous, and to encourage participation, eight EUR 50 gift cards were raffled off as incentives. Ethical approval was granted from the Ethics Committee for Research Involving Human Subjects (CEISH) of the UPV/EHU (M10_2021_185). The participation rate was 1.0% (422/42,200).

Of the 422 respondents, 28 were excluded due to missing responses (e.g., “Don’t know” or “Refused”) to any of the Food Insecurity Experience Scale (FIES) questions (items). The final data set consisted of 394 university students. The characteristics of the excluded participants are summarized in Table S1. It should be noted that the characteristics of the excluded subjects did not differ much from those of the included subjects, except for the percentage of immigrants, which was higher among those excluded than among those who remained in the study (19.4% vs. 6.5%, *p* = 0.015). To ensure representativeness of the UPV/EHU university students’ population, all results were weighted by age and field of education, using weighting coefficients derived from the list of students enrolled during 2021−2022. Information on the population of the UPV/EHU, the theoretical sample achieved, the real sample obtained, the participation rate by field of education and age, and the weighting coefficient assigned to each participant have already been described [24]. Study conduct and reporting comply with the CROSS checklist (Table S2) [26].

### 2.2. Measures

The questionnaire consisted of 70 items and was developed in English in collaboration with all partners of the FINESCOP consortium [24]. A pilot study was carried out in five of the nine participating universities in the FINESCOP project (including the UPV/EHU) before the final version of the questionnaire was approved. The pilot included small convenient samples of college/university students. At the UPV/EHU, a sample of 37 students who responded to the Spanish version and 37 students who answered to the Basque version were analyzed. Internal consistency was evaluated for two of the subsections: socioeconomic and educational factors. The Cronbach’s α results for socioeconomic and educational items in the Spanish version were 0.83 and 0.79, respectively; in the Basque version, they were 0.78 and 0.77, respectively.

In the present manuscript, the following results were provided: demographic variables (sex, age, and migration status, if applicable); socioeconomic variables (ELoP/Lg; ES, that is, number of hours worked before the pandemic, if applicable; changes in ES during the pandemic; changes in the main source of income during the pandemic); FI; health outcomes (current body weight and height, self-reported physical health (SRPH) and self-reported psychological health (SRPsH) before and during the pandemic); and lifestyle factors (vegetable and fruit intake and physical exercise (PE)) during the pandemic.

All questions were adapted from the questionnaires developed and used by Owens et al. [16] and Mahdy et al. [27], except for migration status [28] and parental educational level [29]. FI was measured using the 8-item FIES, evaluating people’s access to adequate food [30]. For analysis, this variable was further dichotomized into two levels: food secure and mild FI (FI_mild_), and moderate/severe FI (FI_mod+sev_). Scores ranging between 1 and 2 were considered FI_mild_, scores between 3 and 5 were considered moderate FI (FI_mod_), and scores equal to or greater than 6 were classified as severe FI (FI_sev_). This classification was determined after testing the validity and reliability of the FIES, during which one item was omitted in the equating process (see the Section 3).

Regarding health outcomes, the BMI was calculated using self-reported weight and height data and classified according to WHO criteria [31]. Participants also reported their SRPH and SRPsH before and during the COVID-19 pandemic using an adaptation of the visual analogue scale (from 0 to 100, with 0 being the worst and 100 the best) of the EuroQuol five-dimensional questionnaire (EQ-5D) [32]. To present the descriptive results of these variables, tertiles were used to deeply understand the distribution and dispersion of data based on previous studies [33,34]. Lifestyle data during the pandemic were collected through a food frequency questionnaire based on those developed by other authors [35,36,37] and a questionnaire of PE [36].

### 2.3. Statistical Analysis

General data were analyzed using IBM Statistical Package for the Social Sciences (SPSS) for Windows, version 28.0 (IBM Corp., Armonk, NY, USA), while the FIES data were analyzed using the open source software RStudio (version 2023.03.1) and FAO’s Excel Template [38]. The Rasch model was used to estimate FI, following FAO recommendations [39]. To implement this model, cases with “Don’t know” or “Refused” responses, as well as extreme raw scores (0 and 8), were excluded from the statistical validation process. After pre-processing, 81 eligible cases (respondents without missing data, that is, without codes for “Don’t know” or “Refused” responses and with raw scores between 1 and 7) were included. The psychometric properties of the FIES were examined using Rasch modeling, examining item fit statistics, overall model fit, and the residual correlation matrix [30,39,40,41].

Descriptive statistics were presented as mean and standard deviation (SD) for continuous data and as percentage for categorical variables. Differences in continuous variables were assessed using the Mann–Whitney U test, given that the variables were not normally distributed (because data were weighted). Categorical variables were analyzed using the Chi-square test. To facilitate the analysis of associations between factors and FI, some variables were dichotomized, including ELoP/Lg (lower than tertiary education versus tertiary education), changes in ES (worsening or not), changes in the main source of income (decreasing or not) during the pandemic, and BMI (normal weight versus Ov/Ob).

Linear regression analyses were conducted to evaluate the association between FI and health outcomes. Results were presented as odds ratios (ORs) with 95% CI. The analysis involved three nested models with different blocks of covariates. Model 1 adjusted only for control variables (sex, age, and migration status), whilst Model 2 included SES (ELoP/Lg and ES). The employment variable was categorized as follows: 0 = not working; 1 = working ≤20 h/week; and 2 = working >20 h/week. The fully adjusted model (Model 3) included variables from Model 2, plus additional lifestyle variables. To satisfy the normality assumptions of the residuals of the linear regression model, the reciprocal transformation was used. Multicollinearity among independent variables was assessed using tolerance and variance inflation factors before interpreting the final output. No variables needed to be excluded. Participants with missing data on the covariates were grouped in a separate category for each; therefore, the analysis was run on the full sample size.

Finally, a bias-corrected bootstrapping analysis (with 10,000 resampling and 95% CI) was performed using the SPSS Process Macro to verify the mediating effects of two indicators of SES. This mediation analysis was based on regression-based path analysis [42,43] and was selected because it is a distribution-independent technique. The two indicators of SES used were the ELoP/Lg and the ES. The first indicator was selected based on its association with students’ dietary patterns in previous studies [44]. Regarding the second indicator, ES was recategorized into two groups [45,46,47]: not working or working ≤20 h/week versus working >20 h/week. For each of the tests, sex, age, and migration were controlled. All tests were two-sided, and *p*-values below 0.05 were considered statistically significant.

## 3. Results

### 3.1. Validation of FIES Data and Prevalence of Food Insecurity

When Rasch modeling was used to statistically validate the FIES data, the distribution of affirmative responses for each item showed that the item FEWFOOD (having to eat only a few kinds of food) was the most frequently reported (56.8%), followed by the item ATELESS (eating less due to lack of resources, 49.4%), while WHLDAY (not eating anything for a whole day because of a lack of money or other resources) was the least reported (3.7%) (Table S3). Most respondents had raw scores of 1 (42%), 2 (27.2%), and 3 (13.6%) (Figure S1).

The item severity parameters showed that FEWFOOD had the lowest severity, followed by ATELESS and WORRIED (being worried about not having enough food to eat) (Table S4). The item WHLDAY had the highest severity. Infit statistics showed that all items were within the acceptable range (0.7–1.3), except for RANOUT (was there a time when you ran out of food?) and WHLDAY, which were slightly above or below that range, respectively, yet still within 0.5 to 1.5, indicating reasonable model fit. All outfits were below 2, showing no significant outliers or unexpected response patterns in the data.

The Rasch reliability coefficient was 0.61, which is slightly below the acceptable range (≥0.7). According to the residual correlation matrix, there was no evidence of multidimensionality in the FIES items, as the correlation across items was within the acceptable range of 0.4. To ensure comparable FI prevalence estimates, the relevant output from RM.weights was inserted into the FAO’s Excel template. Graphical representation of the item parameters showed that the item ATELESS was the furthest away from the diagonal line, with the highest absolute difference (0.96) compared to all other items (Figure S2). The correlation between item parameters of the two scales was 91.2%. As this item (ATELESS) was considered unique, it was excluded from the equation, which increased the correlation between the common items to 94.7%. This resulted in a prevalence rate of 15.7% FI_mild_, 3.3% FI_mod+sev_, and 0.8% severe FI (FI_sev_).

### 3.2. Participant Characteristics

As for the sample characteristics, the majority of the sample were women, and most had parents or guardians with tertiary education (Table 1). FI was significantly associated with being female, older age, immigrant status, lower parental education, working >20 h per week, experiencing worsening in ES, lower vegetable and fruit intake, and reduced PE compared to those who had food security (*p* < 0.001). Most participants were classified as normal weight (80.5%), with 13.1% as Ov/Ob and 6.4% as underweight. The results of self-reported health are shown in Table 2. A summary of the bivariate analysis of demographic and socioeconomic characteristics and lifestyle by FI status and health outcomes is provided in Table S5.

### 3.3. Association Between Food Insecurity, Overweight/Obesity, and Self-Reported Health Status: Socioeconomic Status as a Mediator

The association between FI and health outcomes is shown in Table S6. Most students with FI had Ov/Ob, reported lower SRPH scores, and experienced a greater worsening in both SRPH and SRPsH (*p* < 0.001). These findings were supported by the adjusted linear regression models (Table 3). The associations that remained significant after corrections for multiple testing were those related to BMI, SRPH, and changes in SRPsH and SRPH (except for this last variable in Model 3). Table 4 and Figure S1 indicate the multiple mediation models that include both ELoP/Lg and ES as mediators of the relationship between FI and health outcomes. Unstandardized coefficients showed a significant total association between FI and the BMI (B = 1.85, 95% CI = 0.14–3.56, *p* < 0.05), SRPH (B = −14.85, 95% CI = −24.93–−4.78, *p* < 0.05) and SRPsH (B = −21.38, 95% CI = −32.90–−9.86, *p* < 0.001), in addition to a significant association with ELoP/Lg (a_1_ = −1.05, *p* < 0.05 for the BMI regression; and a_1_ = −0.86, *p* < 0.05 for both the SRPH and SRPsH regressions). These differences in a_1_ are due to different sample sizes, to the fact that there were more missing cases for the BMI variable than for the psychometric variables.

Furthermore, a significant association between ELoP/Lg and BMI was identified (b_1_ = −0.27, *p* < 0.05). However, the direct association was no longer significant when the relationship between the SES and BMI were controlled, indicating total mediation. For SRPH and SRPsH, mediation turned out to be partial, since the direct association remained to be significant when the SES were controlled. According to the mediation percentages, the ELoP/Lg variable can explain 15.3% of the association between FI and the BMI.

## 4. Discussion

This study sought to assess the prevalence of FI among a sample of university students, examine its relationship with multiple health outcomes, and determine whether the SES mediated these relationships. The results of the analysis suggest that a significant proportion of the population experienced FI. Moreover, the findings highlight the impact of FI on various health outcomes, as well as the mediating role of SES in some of these associations.

The prevalence of FI (including FI_mild_ and FI_mod+sev_) in this population (19.0%) is consistent with previous research (19.0–62.8%) conducted across both undergraduate and postgraduate students [16,48,49,50,51]. It is important to note that in the statistical validation of FIES data, rather low reliability was observed when differentiating between distinct strata (food insecure and secure). This may have to do with the relatively low levels of FI reported in the present study, particularly compared to other investigations involving undergraduate or postgraduate students. Continued evaluation and refinement of the FIES items, along with longitudinal studies, will be essential for enhancing the reliability and validity of FI measurements in the study setting. In any case, the prevalence of FI in the current sample was comparable to the reported in U.S. college students in 2019 (19%) [48] and to that estimated amongst European university students during the pandemic [22,23].

At the same time, the rates of FI observed in this study were higher than those reported in Spanish households (~13%) during the COVID-19 pandemic using the FIES [52]. In this regard, prior to the COVID-19 pandemic, studies consistently showed that college students had higher FI rates than non-student U.S. households [48,53,54]. In addition, as observed by other authors [55], in the present investigation, FI was more prevalent among women than men, which may be linked to gender norms and structural discrimination [56]. Similarly to previous studies [16,45,50], the factors representing dimensions of SES were related to FI. These results illustrate the challenges that students of lower SES face in meeting their basic needs, despite working, budgeting, and utilizing available benefits [57]. One institutional barrier for the UPV/EHU students may have been limited access to financial aid. Although the Basque Government provides scholarships to students to pay for tuition and transportation if necessary, having a low income is not the only criterion; academic performance is also required to retain the scholarship. A decline in academic performance during the pandemic may have justified the loss of this economic support, potentially contributing to FI. It should also be noted that the rising cost of college attendance has outpaced the financial aid that students receive [58]; coupled with the shifting demographics of university enrolment, these financial constraints have created a more economically vulnerable student body.

Regarding the associations between FI and health outcomes, students with FI reported higher prevalence of Ov/Ob, poorer self-rated health, and worsening health during the pandemic compared to those who were food secure. This association remained significant after controlling for sociodemographics, economic status, and health behaviors. These findings are consistent with other studies which investigate the consequences of FI for college and university students, both in terms of obesity [59] and worse self-reported physical and mental health [49,60,61]. In addition, the results of this work suggest that FI may act as a stressor that impacts health, and that this association is, in turn, mediated by SES.

Given these data, it is considered necessary to discuss university FI within the context of economic vulnerability. As Willis [60] suggested, while the provision of food offers a remedy to the material aspect of FI, it leaves intact the hierarchical patterns of food quality, access, and acquirement on campuses, which may also be harmful to student health. It is noteworthy that, in the current study, the FIES items with the highest number of affirmative responses were those related to the lack of variety of foods and healthy and nutritious foods. In this regard, it should be remembered that the lack of dietary diversity has been linked to obesity and subsequent poor health outcomes [62]. Likewise, regarding the poor economic situation at the campus level, Goldrick-Rab et al. [47] argued that while financial aid can help, it is often insufficient to cover living costs. These financial constraints have created a more economically vulnerable student population [63].

Regarding intervention strategies to alleviate FI on campus, two initiatives are worth mentioning as examples. The first, led by the University of California, includes food pantries, food voucher programs, and financial assistance, and it has been shown to effectively reduce FI among students [47]. The second one, the Swipe Out Hunger program, enables students to donate unused food vouchers or meal plan credits to peers facing food insecurity through partnerships with university dining providers, ensuring increased access to food without additional institutional funding [64]. Evaluations of federal and university programs indicate that integrating direct food assistance (e.g., expanded eligibility for food assistance, campus pantries) with financial support (e.g., scholarships, emergency aid) is crucial to effectively reducing FI among college students [65]. In addition, offering workshops, online courses, or cooking demonstrations focused on healthy and affordable food can be an important component of nutrition education [66]. Therefore, given the complexity of FI among university students, institutions should consider adopting comprehensive strategies. These initiatives, when combined, can create a more sustainable and inclusive approach to addressing FI among university students.

### Limitations

This study has several limitations. Firstly, the data rely on self-reported measures, which may be subject to recall and social desirability biases or misinterpretation of the questions. To reduce potential biases in self-reported data, validated measures were used; thus, it was assumed that misclassification bias had a limited effect on the main findings of this study. To enhance reliability, future research should incorporate objective dietary assessments (e.g., food records, 24 h recalls, biomarker analyses), as well as direct anthropometric measurements, for a more accurate evaluation of nutritional status and health outcomes. Combining self-reported and objective measures could offer a more comprehensive understanding of the link between FI, health, and SES. Secondly, it is also worth keeping in mind that treating missing data as a separate category may lead to underestimated standard errors. Therefore, future analyses should include alternative assumptions for missing data, such as sensitivity analysis, to better account for potential biases [67].

Thirdly, SES was measured using ELoP/Lg and ES, but these variables may not fully capture financial constraints affecting students. Therefore, in future studies, other indicators of SES, such as household income, financial aid, or student debt levels, should be included to provide a more comprehensive picture. In addition, a detailed analysis of other factors such as lifestyle behaviors and emotional support may also help clarify the mechanisms underlying the observed associations. Moreover, including additional demographic variables, such as ethnicity or disability status, could offer further insights into the differential impact of FI.

Fourthly, a convenience sample from a single university limits the generalizability of the findings. Participation was voluntary, which may have introduced selection bias. However, the sample was weighted according to age and field of education to account for these discrepancies. Fifthly, the survey response rates were low. A potential explanation could be the timing of our survey. The survey request was emailed to students at the end of the first quadrimester (mid-December) when many students were busy doing homework and/or studying for final exams. Similar response rates have been reported by other investigators assessing FI prevalence in college and university students [68].

Finally, the data are cross-sectional, which prevents the establishment of causal relationships; and the focus on a single university limits the applicability of the findings to broader cultural or educational contexts. However, as part of the FINESCOP project, which includes data from nine universities across Europe, future analyses using this extended data set will provide a more comprehensive perspective on FI among university students, helping to mitigate this limitation. Despite these limitations, this study yields important contributions to the field, particularly given the paucity of research to date on the issue of FI and its impact on health outcomes among European university students. A strength of this study is the assessment of many demographic, socioeconomic, and lifestyle factors, which enabled an extensive description of the study population, adjustment of the analyses, and exploration of a potential mediator.

## 5. Conclusions

The results of this study indicate that FI affected 19% of UPV/EHU students, and it was significantly associated with Ov/Ob, self-rated health, and health deterioration during the pandemic. Moreover, these associations were partly mediated by dimensions of SES, specifically by ELoP/Lg and ES. These findings underscore the need for institutional and governmental policies aimed at supporting students’ basic needs and ensuring food security in higher education settings. These policies could include developing and implementing programs that combine direct food assistance (e.g., expanded eligibility for food assistance, campus pantries) with financial support (e.g., scholarships, emergency aid), as well as education in healthy eating habits. For future research, studies with a longitudinal approach would be recommended to systematically monitor FI, examine causal relationships, and enhance robustness. Furthermore, exploring the mediating effect of SES through qualitative interviews or mixed-methods approaches could offer a deeper understanding of students’ lived experiences and coping strategies.

## Figures and Tables

**Table 1:** Sample characteristics: overall, by food insecurity, and overweight/obesity (*n* = 394).

Variable, % ^1^ or Mean (SD)	Overall	FI Status ^2^			Overweight/Obesity		
		None ^3^	Moderate and Severe	*p* ^4^	No ^5^	Yes	*p* ^4^
*n* ^6^	42,135	40,755	1381		31,486	5122	
Demographic variables							
Sex ^7^				<0.001			<0.001
Female	70.3	96.1	3.9		89.3	10.7	
Male	29.7	98.0	2.0		77.4	22.6	
Age	22.8 (5.6)	22.8 (5.6)	24.6 (4.2)	<0.001	22.6 (5.0)	24.1 (7.7)	<0.001
Immigrant				<0.001			<0.001
No	93.5	97.2	2.8		87.0	13.0	
Yes	6.5	90.0	10.0		72.5	27.5	
Socioeconomic variables							
ELoP/Lg ^8^				<0.001			<0.001
<Tertiary education	40.8	94.8	5.2		80.9	19.1	
Tertiary education	59.2	98.0	2.0		89.4	10.6	
Decrease in the main source of income				<0.001			0.581
No	85.6	97.8	2.2		86.8	13.2	
Yes	14.4	91.2	8.8		86.5	13.5	
ES before the pandemic ^9^, h/week				<0.001			<0.001
Unemployed, 0	68.3	97.1	2.9		84.1	15.9	
Employed, ≤20	18.6	96.6	3.4		88.9	11.1	
Employed, >20	13.1	94.8	5.2		91.2	8.8	
Worsening in employment status during the pandemic				0.003			<0.001
No	69.2	96.9	3.1		85.4	14.6	
Yes	30.8	95.9	4.1		91.1	8.9	
Lifestyle during the pandemic (score)							
Vegetable intake (1–8) ^10^	4.4 (1.5)	4.4 (1.5)	3.3 (1.3)	<0.001	4.4 (1.5)	4.2 (1.6)	<0.001
Fruit intake (1–8) ^10^	4.9 (2.1)	4.9 (2.1)	3.6 (1.3)	<0.001	4.8 (2.1)	4.7 (1.8)	<0.001
Exercise (1–7) ^11^	4.5 (1.8)	4.5 (1.8)	3.4 (2.1)	<0.001	4.6 (1.8)	4.2 (1.9)	<0.001

Abbreviations: FI, food insecurity. Note: ^1^ valid percentage; ^2^ scores ranging between 1 and 2 were considered mild FI, between 3 and 5 moderate FI, and equal to or greater than 6 severe FI; ^3^ included: food security and mild food insecurity; ^4^ Chi-square test or Mann–Whitney U test; ^5^ included: only normal weight; ^6^ This table shows the results of the sample made up of 394 university students, but these results were weighted according to age and field of education, using weighting coefficients provided by the list of students enrolled during 2021−2022 (data provided by the Vice-Rectorate of Digital Transformation and Communication of the UPV/EHU); ^7^ The rest of the participants answered “don’t know/don’t answer” or “non-binary”; ^8^ The highest educational level of parents or legal guardians who have achieved the highest educational level; ^9^ included: employed full-time or part-time, self-employed, and seasonal and undeclared jobs (excluding unpaid work or internships); ^10^ 1: “Never/seldom”, 2: “<Once/week”, 3: “1–2 times/week”, 4: “3–4 times/week”, 5: “5–6 times/week”, 6: “Once/day”, 7: “2 times/day”, 8: “≥3 times/day”; ^11^ 1: “<Once/month, 2: “Once/month”, 3: “2–3 times/month”, 4: “About once/week”, 5: “2–3 times/week”, 6: “4–6 times/week”, 7: “Every day”.

**Table 2:** Sample characteristics by self-reported health during the COVID-19 pandemic (*n* = 394).

Variable, % ^1^ or Mean (SD)	SRPH ^2,3^			SRPsH ^2,4^			Worsening SRPH			Worsening SRPsH		
	T1	T3	*p* ^5^	T1	T3	*p* ^5^	No	Yes	*p* ^5^	No	Yes	*p* ^5^
*n* ^6^	13,347	19,611		9274	18,156		25,964	15,329		16,120	25,174	
Demographic variables												
Sex ^7^			<0.001			<0.001			<0.001			<0.001
Female	30.7	50.5		22.3	38.9		65.9	34.3		37.4	62.6	
Male	34.3	41.2		23.6	55.6		57.3	42.7		44.2	55.8	
Age	23.4 (5.0)	22.5 (5.6)	<0.001	22.7 (4.1)	23.3 (6.6)	0.074	23.0 (6.0)	22.5 (4.8)	<0.001	23.4 (7.2)	22.5 (4.3)	<0.001
Immigrant			<0.001			<0.001			0.002			<0.001
No	31.4	47.9		23.1	44.3		62.7	37.3		38.7	61.3	
Yes	44.7	42.1		14.0	40.0		65.7	34.3		43.8	56.2	
Socioeconomic variables												
ELoP/Lg ^8^			<0.001			<0.001			<0.001			<0.001
<Tertiary education	36.2	43.0		20.3	48.1		58.6	41.4		45.9	54.1	
Tertiary education	29.9	51.0		24.4	41.4		65.4	34.6		33.2	66.8	
Decrease in the main source of income			<0.001			<0.001			<0.001			0.001
No	28.8	49.7		21.9	44.8		66.8	33.2		38.5	61.5	
Yes	48.0	32.8		31.8	38.1		43.7	56.3		36.0	64.0	
ES before the pandemic ^9^, h/week			<0.001			<0.001			<0.001			0.013
Unemployed, 0	30.0	49.7		19.4	46.0		51.7	48.3		38.7	61.3	
Employed, ≤20	48.7	31.5		31.2	37.1		44.2	55.8		40.5	59.5	
Employed, >20	20.9	58.9		26.0	43.2		63.6	36.4		38.6	61.4	
Worsening in ES during the pandemic			<0.001			<0.001			<0.001			<0.001
No	31.7	47.1		20.5	44.8		63.6	36.4		39.5	60.5	
Yes	46.3	46.1		47.8	31.2		52.2	47.8		16.3	83.7	
Lifestyle during the pandemic (score)												
Vegetable intake (1–8) ^10^	3.9 (1.5)	4.8(1.4)	<0.001	4.3(1.5)	4.4 (1.6)	<0.001	4.7 (1.4)	3.8 (1.5)	<0.001	4.4 (1.5)	4.3 (1.5)	<0.001
Fruit intake (1–8) ^10^	4.3 (2.0)	5.2(2.1)	<0.001	4.5(2.2)	5.1 (2.0)	<0.001	5.1 (2.1)	4.4 (2.0)	<0.001	5.1 (2.0)	4.7 (2.1)	<0.001
Exercise (1–7) ^11^	3.3 (1.7)	5.1(1.5)	<0.001	4.0(1.9)	4.6 (1.8)	<0.001	4.9 (1.6)	3.6 (1.8)	<0.001	4.8 (1.6)	4.3 (1.9)	<0.001

Abbreviations: ELoP/Lg, educational level of parents/legal guardians; ES, employment situation; SRPH, self-reported physical health; SRPsH, self-reported psychological health; T1, first tertile; T3, third tertile. Note: ^1^ valid percentage; ^2^ the rest of the participants were categorized in the second tertile; ^3^ T1 < 70, T2 ≥ 70 and <80, T3 ≥ 80; ^4^ T1 < 50, T2 ≥ 50 and <70, T3 ≥ 70; ^5^ Chi-square test or Mann–Whitney U test; ^6^ This table shows the results of the sample made up of 394 university students, but these results were weighted according to age and field of education, using weighting coefficients provided by the list of students enrolled during 2021−2022 (data provided by the Vice-Rectorate of Digital Transformation and Communication of the UPV/EHU); ^7^ the rest of the participants answered “don’t know/don’t answer” or “non-binary”; ^8^ The highest educational level of parent or legal guardian who achieved the highest educational level; ^9^ included: employed full-time or part-time, self-employed, and seasonal and undeclared jobs (excluding unpaid work or internships); ^10^ 1: “Never/seldom”, “2: <Once/week”, “3: 1–2 times/week”, “4: 3–4 times/week”, “5: 5–6 times/week”, “6: Once/day”, “7: 2 times/day”, “8: ≥3 times/day”; ^11^ 1: “<Once/month, 2: “Once/month”, 3: “2–3 times/month”, 4: “About once/week”, 5: “2–3 times/week”, 6: “4–6 times/week”, 7: “Every day”.

**Table 3:** Association between food insecurity score and health outcomes (*n* = 394).

Health Outcome (*n*) ^1^	Model	B	95% CI for B	*p*	R^2^
BMI (39,091)	1: Adjusted for control variables ^2^	0.24	0.21–0.28	<0.001	0.055
	2: Model 1 + SES ^3^	0.21	0.17–0.24	<0.001	0.070
	3: Model 2 + lifestyle factors ^4^	0.17	0.13–0.20	<0.001	0.073
SRPH (41,294)	1: Adjusted for control variables ^2^	−2.50	−2.72–−2.28	<0.001	0.019
	2: Model 1 + SES ^3^	−2.60	−2.81–−2.38	<0.001	0.037
	3: Model 2 + lifestyle factors ^4^	−1.48	−1.70–−1.26	<0.001	0.127
SRPsH (41,294)	1: Adjusted for control variables ^2^	−5.72	−5.96–−5.48	<0.001	0.055
	2: Model 1 + SES ^3^	−5.70	−5.95–−5.46	<0.001	0.059
	3: Model 2 + lifestyle factors ^4^	−5.19	−5.44–−4.93	<0.001	0.076
Change in SRPH during the pandemic ^5^ (41,294)	1: Adjusted for control variables ^2^	0.82	0.63–1.01	<0.001	0.021
	2: Model 1 + SES ^3^	0.83	0.65–1.02	<0.001	0.025
	3: Model 2 + lifestyle factors ^4^	−0.13	−0.32–0.06	0.188	0.144
Change in the SRPsH during the pandemic ^5^ (41,294)	1: Adjusted for control variables ^2^	4.02	3.80–4.23	<0.001	0.034
	2: Model 1 + SES ^3^	3.90	3.68–4.12	<0.001	0.042
	3: Model 2 + lifestyle factors ^4^	3.68	3.45–3.91	<0.001	0.073

Abbreviations: B, unstandardized coefficient; SES, socioeconomic status; SRPH, self-reported physical health; SRPsH, self-reported psychological health. Note: ^1^ this table shows the results of the sample made up of 394 university students, but these results were weighted according to age and field of education, using weighting coefficients provided by the list of students enrolled during 2021–2022 (data provided by the Vice-Rectorate of Digital Transformation and Communication of the UPV/EHU); ^2^ adjusted for sociodemographic characteristics (age, sex, immigration); ^3^ adjusted for covariates in Model 1 and socioeconomic status (based on the educational level of parents/legal guardians and employment situation); ^4^ adjusted for covariates in Model 2 and lifestyle factors (vegetable and fruit intake and physical exercise); ^5^ differences between values before and during the pandemic (SRPH or SRPsH_before the pandemic_ − SRPH or SRPsH_during the pandemic_). Differences with the corresponding sign were used; that is, the differences have not been defined in absolute terms.

**Table 4:** Relationship between food insecurity and health outcomes mediated by socioeconomic status (*n* = 394).

Dependent Variable (*n*) ^1^	Model ^2^	Effect	95% CI	*p*	% Mediated
BMI (39,091)	Direct effect	1.60	−0.11–3.31	NS	
	Indirect effect	0.25	−0.17–0.75	<0.05 ^3^	
	ELoP/Lg	0.28	−0.02–0.72	<0.05 ^3^	0.153
	ES	−0.03	−0.27–0.13	<0.05 ^3^	0.017
	Total effect	1.85	0.14–3.56	0.034	
SRPH (41,294)	Direct effect	−14.37	−24.52–−4.23	0.006	
	Indirect effect	−0.48	−2.81–1.41	NS	
	ELoP/Lg	−0.62	−2.62–0.80	<0.05 ^3^	0.042
	ES	0.13	−0.75–1.33	NS	0.009
	Total effect	−14.85	−24.93–−4.78	0.004	
SRPsH (41,294)	Direct effect	−20.10	−31.66–−8.53	<0.001	
	Indirect effect	−1.28	−3.98–0.70	<0.05 ^3^	
	ELoP/Lg	−1.01	−3.65–0.45	<0.05 ^3^	0.050
	ES	−0.27	−1.96–1.29	NS	0.012
	Total effect	−21.38	−32.90–−9.86	<0.001	
Change in SRPH during the pandemic ^4^ (41,294)	Direct effect	4.72	−3.42–12.86	NS	
	Indirect effect	−0.15	−1.71–1.02	NS	
	ELoP/Lg	−0.16	−1.57–0.81	<0.05 ^3^	0.036
	ES	0.01	−0.69–0.73	<0.05 ^3^	0.002
	Total effect	4.57	−3.50–12.63	NS	
Change in the SRPsH during the pandemic ^4^ (41,294)	Direct effect	5.72	−4.78–15.83	NS	
	Indirect effect	0.57	−0.94–2.50	NS	
	ELoP/Lg	0.43	−0.78–2.28	NS	0.072
	ES	0.14	−0.82–1.31	NS	0.022
	Total effect	6.09	−4.13–16.31	NS	

Abbreviations: B, unstandardized coefficient; ELoP/Lg, educational level of parents/legal guardians; ES, employment status; NS, not significant; SRPH, self-reported physical health; SRPsH, self-reported psychological health. Note: ^1^ this table shows the results of the sample made up of 394 university students, but these results were weighted according to age and field of education, using weighting coefficients provided by the list of students enrolled during 2021−2022 (data provided by the Vice-Rectorate of Digital Transformation and Communication of the UPV/EHU); ^2^ model adjusted for sociodemographic characteristics (age, sex, immigration) and mediated by socioeconomic status (based on the educational level of parents/legal guardians and employment status); ^3^ SPSS Process Macro does not estimate the *p*-value, but it was deduced that by not including the CI the unit said value was significant; ^4^ differences between values before and during the pandemic (SRPH or SRPsH_before the pandemic_ − SRPH or SRPsH_during the pandemic_). Differences with the corresponding sign were used; that is, the differences have not been defined in absolute terms.

## Data Availability

Data are to be made available only via request to the corresponding author. Data will be provided only after the acceptance and signature of a formal data-sharing agreement.

## Supplementary Materials

The following supporting information can be downloaded at https://www.mdpi.com/article/10.3390/nu17081314/s1, Table S1: Demographic and socioeconomic characteristics of the excluded participants; Table S2: Checklist for Reporting Of Survey Studies (CROSS); Table S3: FIES questions (item) and affirmative answers; Table S4: Food Insecurity Experience Scale (FIES) item statistics; Table S5: Summary of the bivariate analyses of demographic and socioeconomic characteristics and lifestyle by food insecurity and health outcomes; Table S6. Food insecurity status by health outcomes; Figure S1: Distribution of raw scores in the subsample in which the Rasch model was applied (*n* = 81); Figure S2: First equating scenario (all FIES items determined to be common); Figure S3: Multiple mediation models.
