# Supplementary material for: Food Insecurity, Health, and Socioeconomic Status: Results from the University of the Basque Country, Spain
Source: Nutrients. 2025 Apr 10;17(8):1314. doi: 10.3390/nu17081314 (PMC12030259; doi:10.3390/nu17081314)
Supplement: Supplementary file 1 [file nutrients-17-01314-s001.zip › nutrients-3564735-supplementary.pdf]

Supplementary Material

This supplementary material has been provided by the authors to give readers additional information about their work.

Supplement to: García-Iruretagoyena L, Martinez-Perez N, Colen L, Baeta M, Olalde I, Torheim LE, Arroyo-Izaga M. Food Insecurity, Health and Socioeconomic Status: Results from the University of the Basque Country, Spain. *Nutrients* **2025**

Corresponding authors: lgarcia.ir@gmail.com (L.G.-I.); naiara.martinez@ehu.eus (N.M.-P.); marta.arroyo@ehu.eus (M.A.-I.)

Table S1. Demographic and socioeconomic characteristics of the excluded participants.

Variables, % [1] or mean (SD)	Excluded participants (*n* = 28)
Demographic variables	
Sex [2]	
Female	69.0
Male	31.0
Age, years	22.5 (3.1)
Immigrant [3]	
No	80.6
Yes	19.4
Socioeconomic variables	
ELoP/Lg [4]	
< Tertiary education	43.3
Tertiary education	56.7
Decrease in the main source of income	
No	78.3
Yes	21.7
Employed before the pandemic [5], h/wk	
Unemployed, 0	61.3
Employed, ≤ 20	25.8
Employed, > 20	12.9
Worsening in ES during the pandemic	
No	75.9
Yes	24.1
Lifestyle during the pandemic (score)	
Vegetable intake (1–8) [6]	4.5 (1.6)
Fruit intake (1–8) [6]	4.3 (1.9)
Exercise (1–7) [7]	3.8 (1.8)

Abbreviations: ELoP/Lg, educational level of parents/legal guardians; ES, employment situation. Note: [1] valid percentage; [2] the rest of participants answered "don't know/don't answer" or "non-binary"; [3] the rest of participants answered "don't know/don't answer"; [4] the highest educational level of parent or legal guardian who achieved the highest educational level; [5] employed full-time or part-time, self-employed, and seasonal and undeclared jobs (excluding unpaid work or internships); [6] 1: "Never/seldom", 2: "<Once/week", 3: "1–2 times/week", 4: "3–4 times/week", 5: "5–6 times/week", 6: "Once/day", 7: "2 times/day", 8: "≥3 times/day"; [7] 1: "<Once/month, 2: "Once/month", 3: "2–3 times/ month", 4: "About once/week", 5: "2–3 times/week", 6: "4–6 times/week", 7: "Every day".

Table S2. Checklist for Reporting Of Survey Studies (CROSS).

Section/topic	Item	Item description	Reported on
		Title and abstract	
Title and abstract	1a	State the word "survey" along with a commonly used term in title or abstract.	Abstract
	1b	Provide an informative summary in the abstract, covering background, objectives, methods, findings/results, interpretation/discussion, and conclusions.	Abstract
		Introduction	
Background	2	Provide a background about the rationale of study, what has been previously done, and why this survey is needed.	Introduction, paragraphs 1–4
Purpose/aim	3	Identify specific purposes, aims, goals, or objectives of the study.	Introduction, paragraph 5
		Methods	
Study design	4	Specify the study design in the methods section with a commonly used term (e.g., cross-sectional or longitudinal).	Methods, Study sample, paragraph 1
Data collection methods	5a	Describe the questionnaire (e.g., number of sections, number of questions, number and names of instruments used).	Methods, Measures, paragraphs 1
	5b	Describe all questionnaire instruments that were used in the survey to measure particular concepts. Report target population, reported validity and reliability information, scoring/classification procedure, and reference links (if any).	Methods, Measures, paragraphs 2–4
	5c	Provide information on pretesting of the questionnaire, if performed (in the article or in an online supplement). Report the method of pretesting, number of times questionnaire was pre-tested, number and demographics of participants used for pretesting, and the level of similarity of demographics between pre-testing participants and sample population.	Methods, Measures, paragraph 1
	5d	Questionnaire if possible, should be fully provided (in the article, or as appendices or as an online supplement).	Methods, Measures, paragraph 1
Sample characteristics	6a	Describe the study population (i.e., background, locations, eligibility criteria for participant inclusion in survey, exclusion criteria).	Methods, Study sample, paragraphs 1–3
	6b	Describe the sampling techniques used (e.g., single stage or multistage sampling, simple random sampling, stratified sampling, cluster sampling, convenience sampling). Specify the locations of sample participants whenever	Methods, Study sample, paragraph 1

Supplement to: García-Iruretagoyena L, Martinez-Perez N, Colen L, Baeta M, Olalde I, Torheim LE, Arroyo-Izaga M. Food Insecurity, Health and Socioeconomic Status: Results from the University of the Basque Country, Spain. *Nutrients* **2025**

		clustered sampling was applied.	
	6c	Provide information on sample size, along with details of sample size calculation.	Methods, Study sample, paragraph 1
	6d	Describe how representative the sample is of the study population (or target population if possible), particularly for population-based surveys.	Methods, Study sample, paragraph 3
Survey administration	7a	Provide information on modes of questionnaire administration, including the type and number of contacts, the location where the survey was conducted (e.g., outpatient room or by use of online tools, such as SurveyMonkey).	Methods, Study sample, paragraph 2
	7b	Provide information of survey's time frame, such as periods of recruitment, exposure, and follow-up days.	Methods, Study sample, paragraph 1
	7c	Provide information on the entry process: –>For non-web-based surveys, provide approaches to minimize human error in data entry.	Not Applicable
		–>For web-based surveys, provide approaches to prevent "multiple participation" of participants.	Methods, Study sample, paragraph 2
Study preparation	8	Describe any preparation process before conducting the survey (e.g., interviewers' training process, advertising the survey).	Methods, Study sample, paragraph 2
Ethical considerations	9a	Provide information on ethical approval for the survey if obtained, including informed consent, institutional review board [IRB] approval, Helsinki declaration, and good clinical practice [GCP] declaration (as appropriate).	Methods, Study sample, paragraph 2
	9b	Provide information about survey anonymity and confidentiality and describe what mechanisms were used to protect unauthorized access.	Methods, Study sample, paragraph 2
Statistical analysis	10a	Describe statistical methods and analytical approach. Report the statistical software that was used for data analysis.	Methods, Statistical Analysis, paragraphs 1–4
	10b	Report any modification of variables used in the analysis, along with reference (if available).	Methods, Statistical Analysis, paragraphs 2–4
	10c	Report details about how missing data was handled. Include rate of missing items, missing data mechanism (i.e., missing completely at random [MCAR], missing at random [MAR] or missing not at random [MNAR]) and methods used to deal with missing data (e.g., multiple imputation).	Methods, Statistical Analysis, paragraphs 1 and 3

	10d	State how non-response error was addressed.	Methods, Study sample, paragraph 3; Discussion, limitations, paragraph 1
	10e	For longitudinal surveys, state how loss to follow-up was addressed.	Not Applicable
	10f	Indicate whether any methods such as weighting of items or propensity scores have been used to adjust for non-representativeness of the sample.	Methods, Study sample, paragraph 3
	10g	Describe any sensitivity analysis conducted.	Not Applicable
Results			
Respondent characteristics	11a	Report numbers of individuals at each stage of the study. Consider using a flow diagram, if possible.	Not Applicable
	11b	Provide reasons for non-participation at each stage, if possible.	Not Applicable
	11c	Report response rate, present the definition of response rate or the formula used to calculate response rate.	Methods, Study sample, paragraph 2
	11d	Provide information to define how unique visitors are determined. Report number of unique visitors along with relevant proportions (e.g., view proportion, participation proportion, completion proportion).	Not Applicable
Descriptive results	12	Provide characteristics of study participants, as well as information on potential confounders and assessed outcomes.	Results, paragraph 4
Main findings	13a	Give unadjusted estimates and, if applicable, confounder-adjusted estimates along with 95% confidence intervals and p-values.	Results, Tables 3 and 4
	13b	For multivariable analysis, provide information on the model building process, model fit statistics, and model assumptions (as appropriate).	Methods, Statistical Analysis, paragraphs 3 and 4
	13c	Provide details about any sensitivity analysis performed. If there are considerable amount of missing data, report sensitivity analyses comparing the results of complete cases with that of the imputed dataset (if possible).	Not applicable
Discussion			
Limitations	14	Discuss the limitations of the study, considering sources of potential biases and imprecisions, such as non-representativeness of sample, study design, important uncontrolled confounders.	Discussion, Limitations
Interpretations	15	Give a cautious overall interpretation of results, based on potential biases and imprecisions and suggest areas for future research.	Discussion, Limitations

Generalizability	16	Discuss the external validity of the results.	Discussion, Limitations
		Other sections	
Role of funding source	17	State whether any funding organization has had any roles in the survey's design, implementation, and analysis.	Financial disclosure
Conflict of interest	18	Declare any potential conflict of interest.	Conflict of interest statement
Acknowledgements	19	Provide names of organizations/persons that are acknowledged along with their contribution to the research.	Acknowledgments

Table S3. FIES questions (item) and affirmative answers.

Item	Standard label	Question wording	%
		**Now I would like to ask you some questions about food. During the last 12 months, was there a time when….**	
1	WORRIED	You were **worried** you would not have enough food toeat because of a lack of money or other resources?	37.0
2	HEALTHY	You were unable to eat **healthy and nutritious food** because of a lack of money or other resources?	34.6
3	FEWFOODS	You ate only a **few kinds of foods** because of lack ofmoney or other resources?	56.8
4	SKIPPED	You had to **skip a meal** because there was not enoughmoney or other resources to get food?	18.5
5	ATELESS	You **ate less** than you thought you should because of alack of money or other resources?	49.4
6	RANOUT	Your household **ran out of food** because of a lack ofmoney or other resources?	9.9
7	HUNGRY	You were **hungry** but did not eat because there was notenough money or other resources?	17.3
8	WHLDAY	You went **without eating for a whole day** because of alack of money or other resources?	3.7

Table S4. Food Insecurity Experience Scale (FIES) item statistics.

Item	Item severity	SE	Infit	Outfit
WORRIED	-0.825	0.271	1.175	1.293
HEALTHY	-0.701	0.276	1.106	1.518
FEWFOOD	-1.669	0.252	0.915	0.844
SKIPPED	0.352	0.347	0.886	0.851
ATELESS	-1.375	0.255	0.945	0.923
RANOUT	1.288	0.442	1.355	1.572
HUNGRY	0.462	0.356	0.816	0.578
WHLDAY	2.468	0.638	0.624	0.116

Abbreviation: SE, standard.

Table S5. Summary of the bivariate analyses of demographic and socioeconomic characteristics and lifestyle by food insecurity and health outcomes.

	Food insecurity [1]	Overweight/ obesity	SRPH (T1)	SRPsH (T1)	Worsening SRPH	Worsening SRPsH
Demographic variables						
Sex						
Female	X					X
Male		X	X	X	X	
Age						
Older	X	X	X			
Younger				X	X	X
Immigrant						
No				X	X	X
Yes	X	X	X			
Socioeconomic variables						
ELoP/Lg [2]						
< Tertiary education	X	X	X		X	
Tertiary education				X		X
Decrease in the main source of income	X		X	X	X	X
Employed before the pandemic [3]						
Unemployed		X				X
≤ 20 h/wk			X	X	X	
> 20 h/wk	X					
Worsening in ES during the pandemic						
No		X				
Yes	X		X	X	X	X
Lifestyle during the pandemic (lower scores)						
Vegetable intake	X	X	X	X	X	X
Fruit intake	X	X	X	X	X	X
Exercise	X	X	X	X	X	X

Abbreviations: ELoP/Lg, educational level of parents/legal guardians; ES, employment situation; SRPH, self-reported physical health; SRPsH, self-reported psychological health; T1, first tertile; X, analyses were performed using Chi-square test or Mann-Whitney U test, significant differences ($p < 0.05$) between categories are highlighted with an X. Note*:* [1] included: moderate and severe food insecurity, Food security and mild food insecurity that is, scores equal to or greater than 3; [2] the highest educational level of parents or legal guardian who achieved the highest educational level; [3] included: employed full-time or part-time, self-employed, and seasonal and undeclared jobs (excluding unpaid work or internships).

Table S6. Food insecurity status by health outcomes.

Variable, % [1] or mean (SD)	FI status [2]		
	None & mild [3]	Moderate & severe	*p* [4]
n [5]	**40,755**	**1381**	
Weight status			
Underweight (BMI < 18.5 kg/m^2)	97.4	2.6	<0.001
Normal weight (BMI = 18.5–25.0 kg/m^2)	98.1	1.9	
Overweight/obesity (BMI ≥ 25.0 kg/m^2)	90.6	9.4	
SRPH during the pandemic	70.0 (21.0)	53.4 (23.5)	<0.001
SRPsH during the pandemic	59.7 (23.5)	39.9 (29.2)	<0.001
Differences in SRPH before and during the pandemic [6]	3.5 (18.4)	10.2 (27.8)	<0.001
Differences in SRPsH before and during the pandemic [6]	10.9 (21.3)	19.6 (28.0)	<0.001

Abbreviations: FI, food insecurity; NS, not significant; SD, standard deviation; SRPH, self-reported physical health; SRPsH, self-reported psychological health. Note: [1] valid percentage; [2] scores ranged between 1 and 2 were considered mild FI, between 3 and 5 moderate FI, and equal to or greater than 6 severe FI; [3] Included: Food security and mild food insecurity; [4] Chi-square test or Mann-Whitney U test; [5] this table shows the results of the sample made up of 394 university students, but these results were weighted according to age and field of education, using weighting coefficients provided by the list of student enrolled during 2021−2022 (data provided by the Vice-Rectorate of Digital Transformation and Communication of the UPV/EHU); [6] differences were defined as before − during. These differences are presented with the corresponding sign were used, that is, the differences have been not defined in absolute terms.

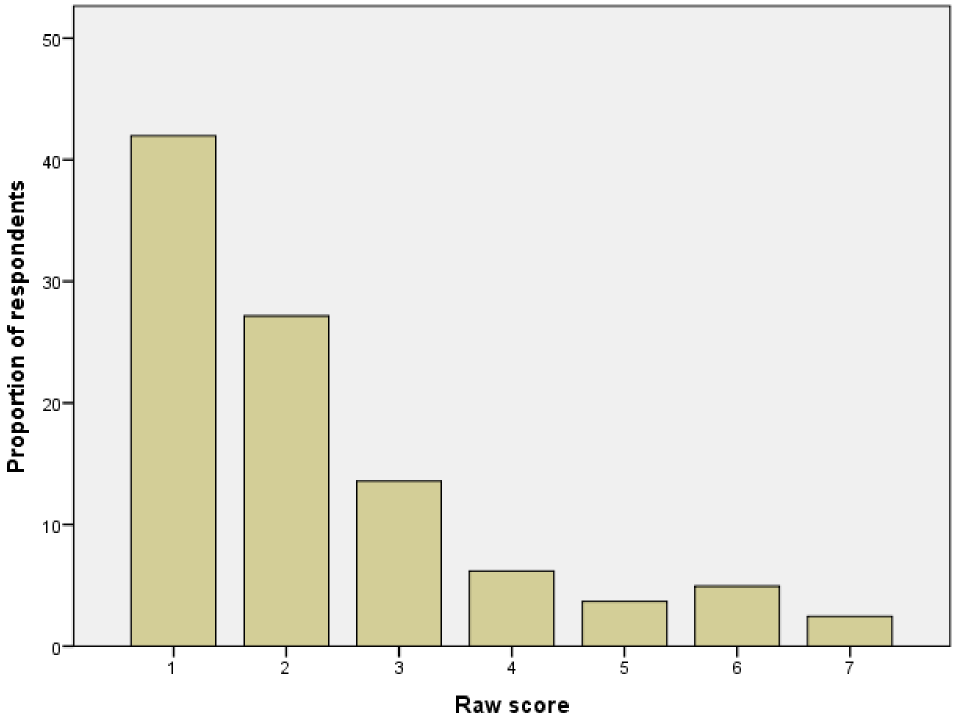

Figure S1. Distribution of raw scores in the subsample in which the Rasch model was applied (*n* = 81).

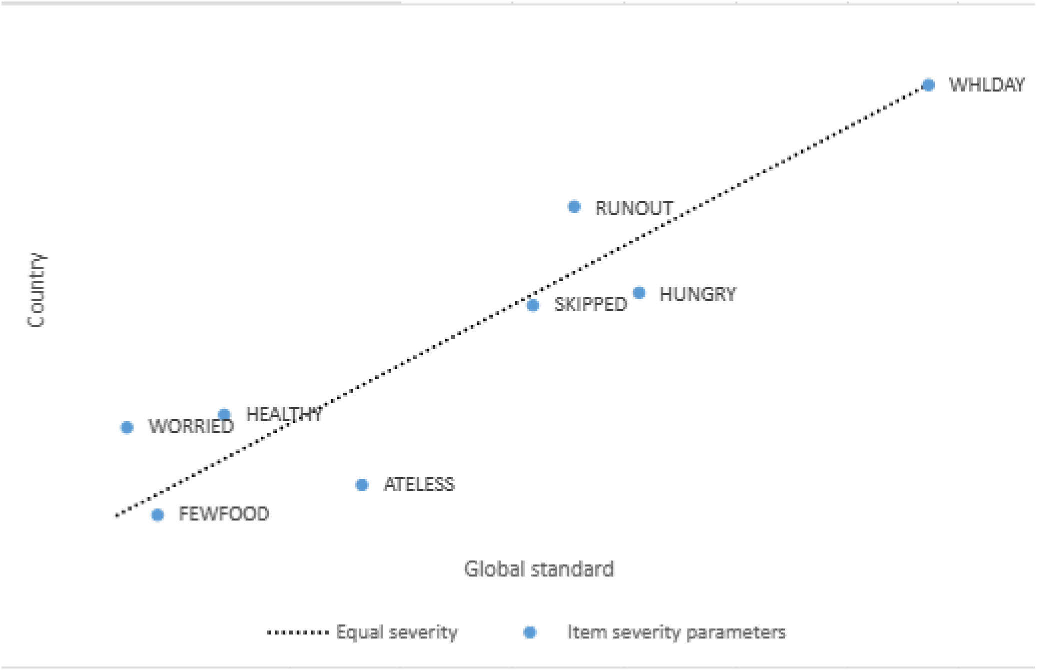

Figure S2. First equating scenario (all FIES items determined to be common).

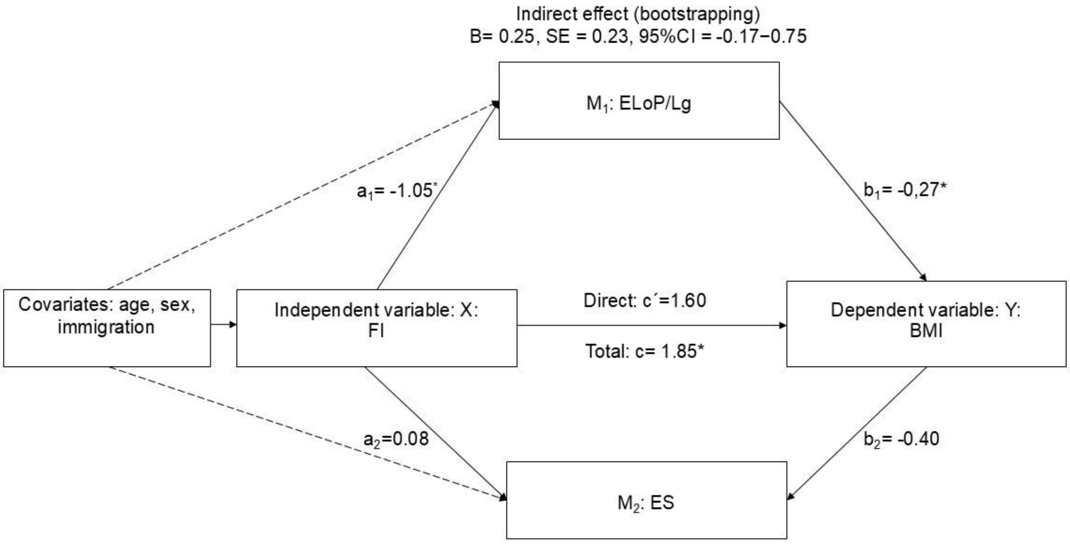
Indirect effect (bootstrapping)
B= 0.25, SE = 0.23, 95%CI = -0.17−0.75
M1: ELoP/Lg
a1= -1.05*
b1= -0,27*
Covariates: age, sex, immigration
Independent variable: X: FI
Direct: c´=1.60
Total: c= 1.85*
Dependent variable: Y: BMI
a2=0.08
b2= -0.40
M2: ES

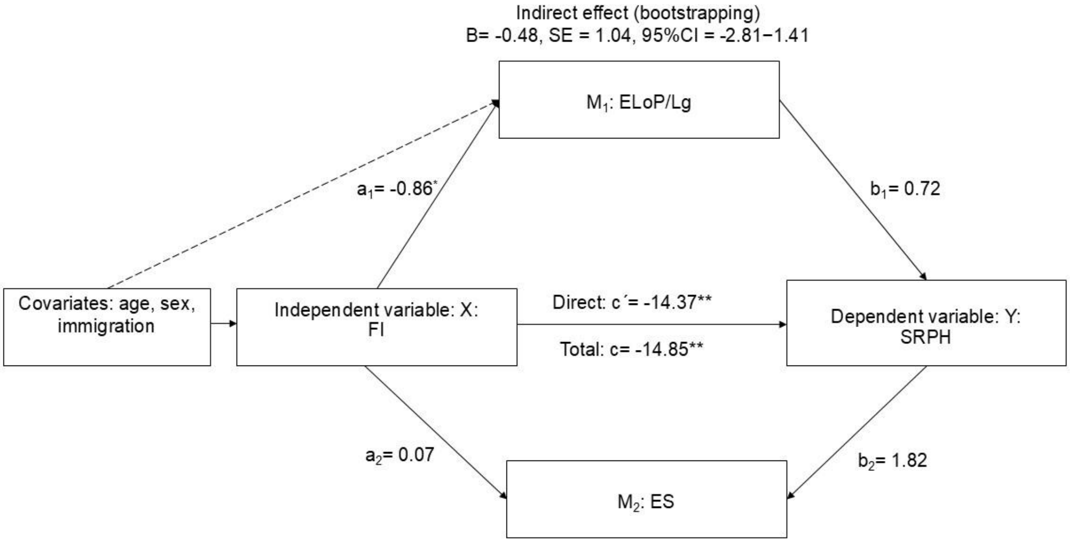
Indirect effect (bootstrapping)
B= -0.48, SE = 1.04, 95%CI = -2.81−1.41
M1: ELoP/Lg
a1= -0.86*
b1= 0.72
Covariates: age, sex, immigration
Independent variable: X: FI
Direct: c´= -14.37**
Total: c= -14.85**
Dependent variable: Y: SRPH
a2= 0.07
b2= 1.82
M2: ES

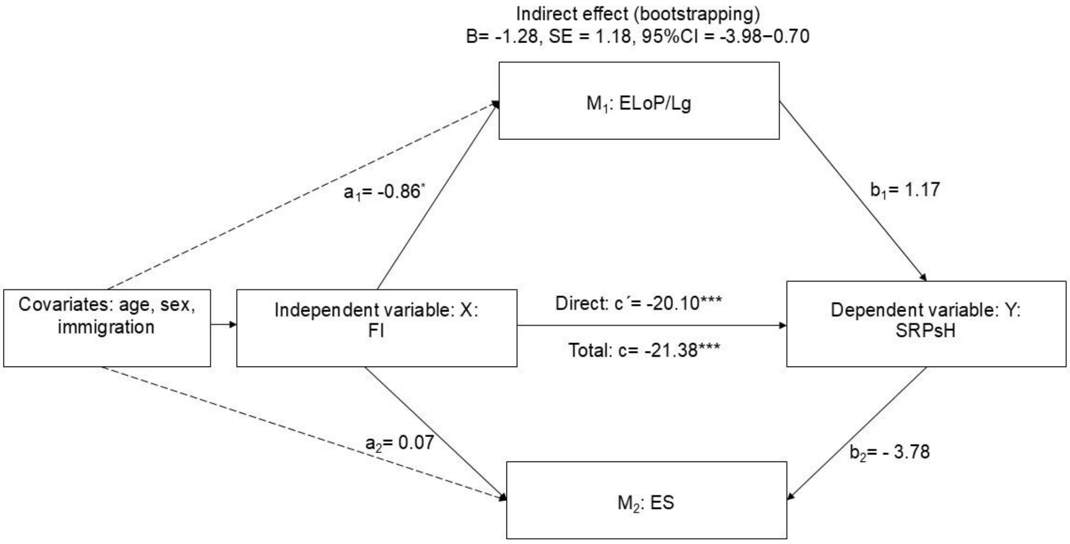
Indirect effect (bootstrapping)
B= -1.28, SE = 1.18, 95%CI = -3.98−0.70
M1: ELoP/Lg
a1= -0.86*
b1= 1.17
Covariates: age, sex, immigration
Independent variable: X: FI
Direct: c´= -20.10***
Total: c= -21.38***
Dependent variable: Y: SRPsH
a2= 0.07
b2= - 3.78
M2: ES

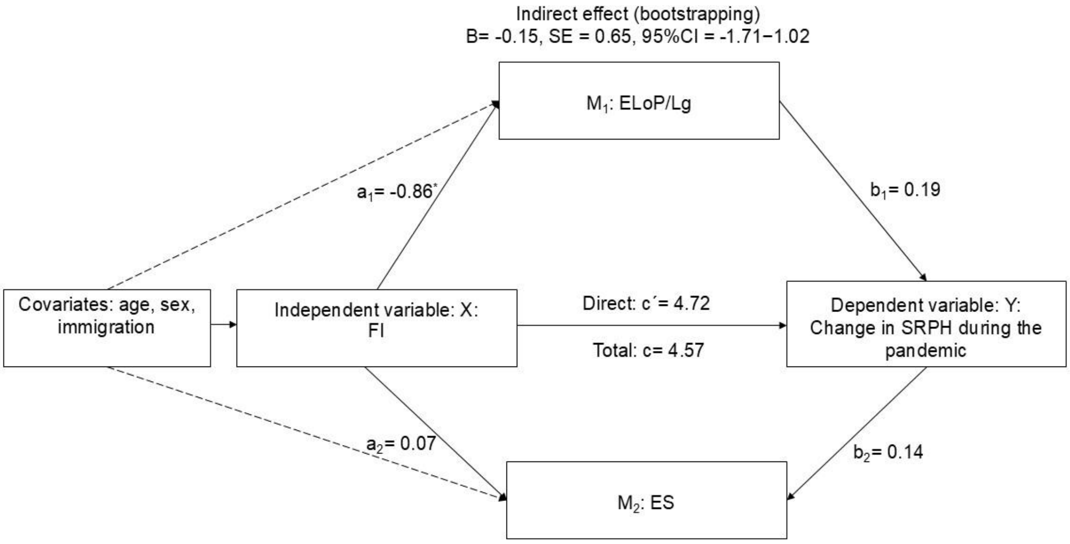
Indirect effect (bootstrapping)
B= -0.15, SE = 0.65, 95%CI = -1.71−1.02
M1: ELoP/Lg
a1= -0.86*
b1= 0.19
Covariates: age, sex, immigration
Independent variable: X: FI
Direct: c´= 4.72
Total: c= 4.57
Dependent variable: Y: Change in SRPH during the pandemic
a2= 0.07
b2= 0.14
M2: ES

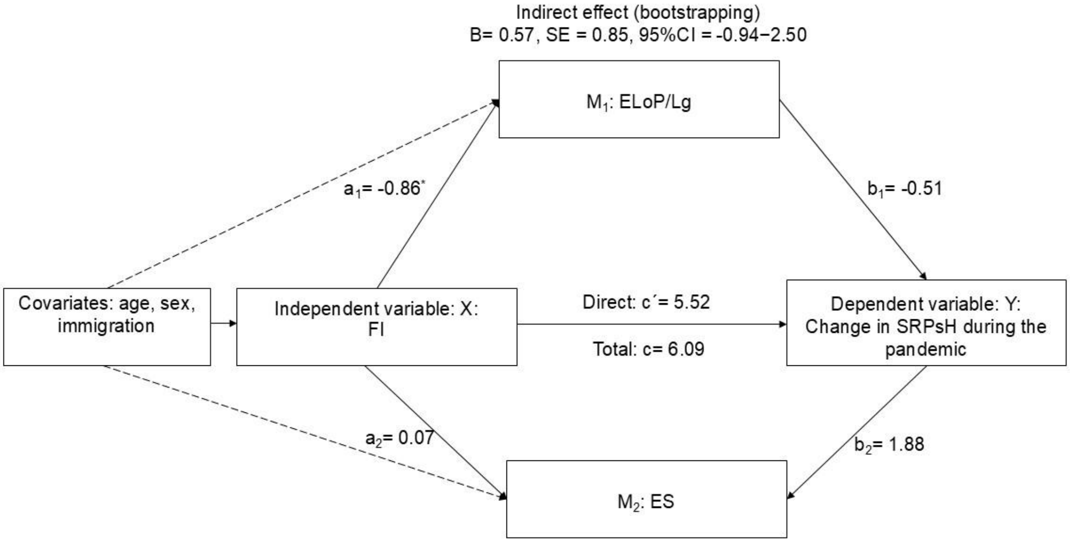

Figure S3. Multiple mediation models. Abbreviations: B, unstandardized coefficient; ELoP/Lg, educational level of parents/legal guardians; ES, employment situation; FI, food insecurity; SE, standard error; SRPH, self-reported physical health; SRPsH, self-reported psychological health. **p*<0.05; ***p*<0.01; ****p*<0.001.
